# Mathematical model and stability of SWCNT- and MWCNT-based nanofluid flow with thermal and chemically reactive effects inside a porous vertical cone

**DOI:** 10.3389/fchem.2024.1463778

**Published:** 2025-01-14

**Authors:** Haihua Xu, Fuad A. Awwad, Emad A. A. Ismail, Waris Khan

**Affiliations:** ^1^ School of Mechanical Engineering, Chongqing Three Gorges University, Chongqing, China; ^2^ Department of Quantitative Analysis, College of Business Administration, King Saud University, Riyadh, Saudi Arabia; ^3^ Department of Mathematics and Statistics, Hazara University, Mansehra, Pakistan

**Keywords:** numerical investigation, dual solution, stability analysis, nanofluid, thermal radiation, solutal boundary layer, chemical performance, heat source

## Abstract

This study investigates the significance of single-walled (SWCNTs) and multi-walled (MWCNTs) carbon nanotubes with a convectional fluid (water) over a vertical cone under the influences of chemical reaction, magnetic field, thermal radiation and saturated porous media. The impact of heat sources is also examined. Based on the flow assumptions, the fundamental flow equations are modeled as partial differential equations (PDEs). Using the appropriate transformation, the PDEs are converted to ordinary differential equations and then solved via RK4 in MATLAB. To confirm the results, a comparison is made with a previously investigated problem, finding good agreement. The emerging dimensionless physical parameters impact on the flow problem is determined through graphs and tables. Analysis reveals a dual solution for the suction and injection parameter. Therefore, stability examination is implemented to confirm a stable solution. The aim of the study is to analyze SWCTs and MWCTs in a vertical cone with stability to establish that only the first solution is reliable. The analysis here signifies that large volume fractions can be substituted to increase the nanofluid movement. The mathematical model and graphical demonstration indicate that the velocity of MWCNTs is higher than SWCNTs. Moreover, the local skin friction, rate of heat transfer, and Nusselt and Sherwood numbers improve with Biot number.

## 1 Introduction

Heat transmission is crucial for both machine performance and material choice. The biological sciences, thermoregulation, bio-heat transfer activities, and blood circulation all greatly benefit from heat transmission. Every biological and mechanical process eventually loses efficiency because it generates heat; regular heat removal from a system is required to maximize its effectiveness. Nanofluids (NFs) are novel substances which can regulate heat conduction. Water, polymerization, engine oil, and various lubricants are examples of common fluids that have lower heating conductivity than solids. Increasing the heat conductivity of such fluids is as simple as adding a certain quantity of solid particles. Nanomaterials are small particles, typically 1–100 nm in size; their resulting fluid is known as “nanofluid”. Choi and Eastman (1995a; 1995b) first recommended the creation of NFs. Xuan and Li (2000) discussed developments in NF heat transmission. To analyze the movement of NFs, Buongiorno (2006) derived a model that included thermophoresis and Brownian motion dynamics. Many singularities, including the reduction or augmentation of the network’s thermal magnitude, the buildup of microchannels, the miniaturization of the entire structure, and minimum clogging, may be better understood using NFs. The electromagnetic dipole impact on Williamson NF under heat was studied by Khan et al. (2020a); they also investigated entropy production on magneto-fluid under robin restrictions with dissipation (Khan et al., 2020b).

Memon et al. (2022) and Abu-Bakr et al. (2022) discuss the larger body of work on heat transmission using NFs. Because of its extensive uses in industry, research, and engineering, heat-mass transfer has attracted much scholarly attention. It is crucial for raising the threshold for final product quality and finds use in ferromagnetic medication targeting and the cooling process of electronic gadgets, among other chemical and pharmacological processes. Hayat et al. (2017a) studied the computational analysis of carbon nanotubes (CNTs) with slip conditions. Hayat et al. (2017b) investigated homogeneous as well as heterogeneous influences in fluid movement with joule heating. Hussain et al. (2022) reported the slip and joule effects in NF over stretched plate. Similarly, Hayat et al. (2019) examined Jeffrey NF in Darcian flow with heat source and melting effects. Other practical uses include insulation, the storage of thermal energy, the extraction and utilization of fossil fuels, the paper sector, food preparation, the inclusion of oxygen, drying porous solids, dialysis treatment, underground energy dissemination, and the restoration of geothermal energy. Peristaltic movement, heat transmission, mass transportation, and chemical processes were all investigated by HinanS et al. (2012).

CNTs have malleable strengths ranging 11–63 GPa and an ultra-high Young’s modulus of roughly 1 TPa—extraordinary mechanical qualities. The elimination of heat is currently of importance to the electronics sector as it strives to achieve higher levels of degenerative power. In order to create future generations of ICs and 3D technological devices, we must determine which materials offer the best heat transfer properties. A CNT is a man-made cylinder made from a rolled-up strip of graphene that can be either a single- or multi-well structure. CNTs with only one hole and a diameter of 0.4–3 nm are called “single-wall carbon nanotubes” (SWCNTs), whereas CNTs with multiple wells and a diameter of 0.4–30 nm are called “multi-wall carbon nanotubes” (MWCNTs). CNTs are employed in a wide variety of industries, including water purification, healthcare, nanotechnology battery production, superconductivity, electronics, recycling, transplantation, solar energy storage, and biological sensors. Using Maxwell hypothesis, Xue (2005) provided a model for thermal conduction that shows the effect of space-dispersed CNTs in a spinning tube. When the liquid heat is 25°C, the heating capacity of the CNTs-based nanofluid is enhanced by up to 30%; at 40°C, the augmentation rises to 79%. Khan (2023a) used ferro nanoparticles to examine the agnetized Williamson NF. The unsteady thermal mass transfer unmagnetized Sutterby NF was studied by Azeem Khan (2022). The radioactive impact in Eyring–Powell fluid comprising gyrotactic microorganisms was described by Khan (2023b). A computational approach was developed for thermally Eyring–Powell fluid exposed to biological convection behavior (Anjum et al., 2022). Waqas et al. (2022) studied the ferromagnetic Casson NF that comprises stratifications in bio-convection movement.

The heat transmission rates of MWCNTs suspended in filtered water across a horizontal heated tube were experimentally investigated by Amrollahi et al. (2010); for a nanotube volume fraction of 0.25%, they found a 33–40% increase in the laminar heat transmission coefficient. The thermal properties of convectional (water) CNT NF was experimentally established to be improved by 100%–250% by Rashmi et al. (2011). Thermophysical characteristics and the general efficacy of MWCNT oil-depending NF movement across helically wrapped tubes was reported by Pakdamana et al. (2012), who also noted an increase in thermal efficiency when the volume friction of suspended CNTs was 0.1%, 0.2%, or 0.4%. Heat transmission along with pressure drop of CNT-convectional (water) NF through a flat cylindrical tube using distilled water as base fluid was inspected by Wang et al. (2013). Using several types of nanoparticles, Akbar et al. (2023a) investigated the peristaltic motion of magnetized tiny fluids. The heat transfer of a magnetized elastic liquid through entropy impact was studied by Akbar et al. (2023b). Using a non-uniform structure, Maraj et al. (2023) investigated NF in peristaltic mobility. Similarly, Akbar et al. (2024) examined thermal properties in microchannels using copper and silver nanotechnology in micropolar liquid. The heat phenomenon in Eyring–Powell fluid across an extensible sheet was observed by Alghamdi et al. (2024).

The fields of biomedical science, optics, magnetic cell segregation, silk float separations, nonlinear optics, heat exhaustion, drug administration, and optical grates are a few applications of magnetic NFs, in which liquid and magnetic characteristics coexist. There has been much research into movement and heat mass transportation using magnetic NFs (Hayat et al., 2011; Chamkha and Rashad, 2014; Sudarsana Reddy and Chamkha, 2016a; Sudarsana Reddy and Chamkha, 2016b; Tasawar et al., 2015). Heatline analysis was provided by Khan et al. (2015) to investigate heat transportation using inclined heated surface containing CuO-water-based NF. Numerical analysis conducted by Selimefendigil and Oztop (2016a) on Al_2_O_3_ and CuO-water-based NF in an vertical heated cavity found that heat transportation upsurges with the volume fraction of nano-size particles. Magnetized mixed conduction heat exchange improvement of CuO-based NF over a lid-driven chamber was studied by Selimefendigil and Oztop (2016b), who discovered that the absolute magnitude of average heat transmission rises from 1 to 100 when the Richardson number grows. Heat transmission was shown to decrease with increasing Hartman number in natural porous chamber flow containing CuO NF (Miroshnichenko et al., 2016; Oztop et al., 2017).

According to Amrollahi et al. (2010), Rashmi et al. (2011), Pakdamana et al. (2012), Wang et al. (2013), and Akbar et al. (2023a), there is no research on the inspiration of radiation effect on the SWCNT and MWCNT NF flowing in an upward cone via porous media with heat source and convective boundary conditions enclosing heat mass transportation with stability analysis. This study aims to investigate the significance of SWCNTs and MWCNTs in convectional fluid (water) over a vertical cone under the influences of chemical reaction, magnetism, thermal radiation, and drenched porous media. The impact of heat is also examined. Based on the flow molds, the fundamental flow characteristics are modeled in term of partial differential equations (PDEs). Using the appropriate transformation, the PDEs are dimentionalized in terms of conventional derivatives (ordinary differential equations) and then solved via RK4 in MATLAB. The present analysis reveals the dual solution for the suction and injection parameter. Therefore, an immovability investigation is conducted to confirm which solution is stable. This study is analyzes SWCTs and MWCNTs in vertical cones with stability to establish that only the first solution is reliable. Solar systems, refinement systems, pharmaceutical materials, and thermal electrodes are the immediate applications of this analysis.

## 2 Mathematical analysis

**FIGURE 1:**
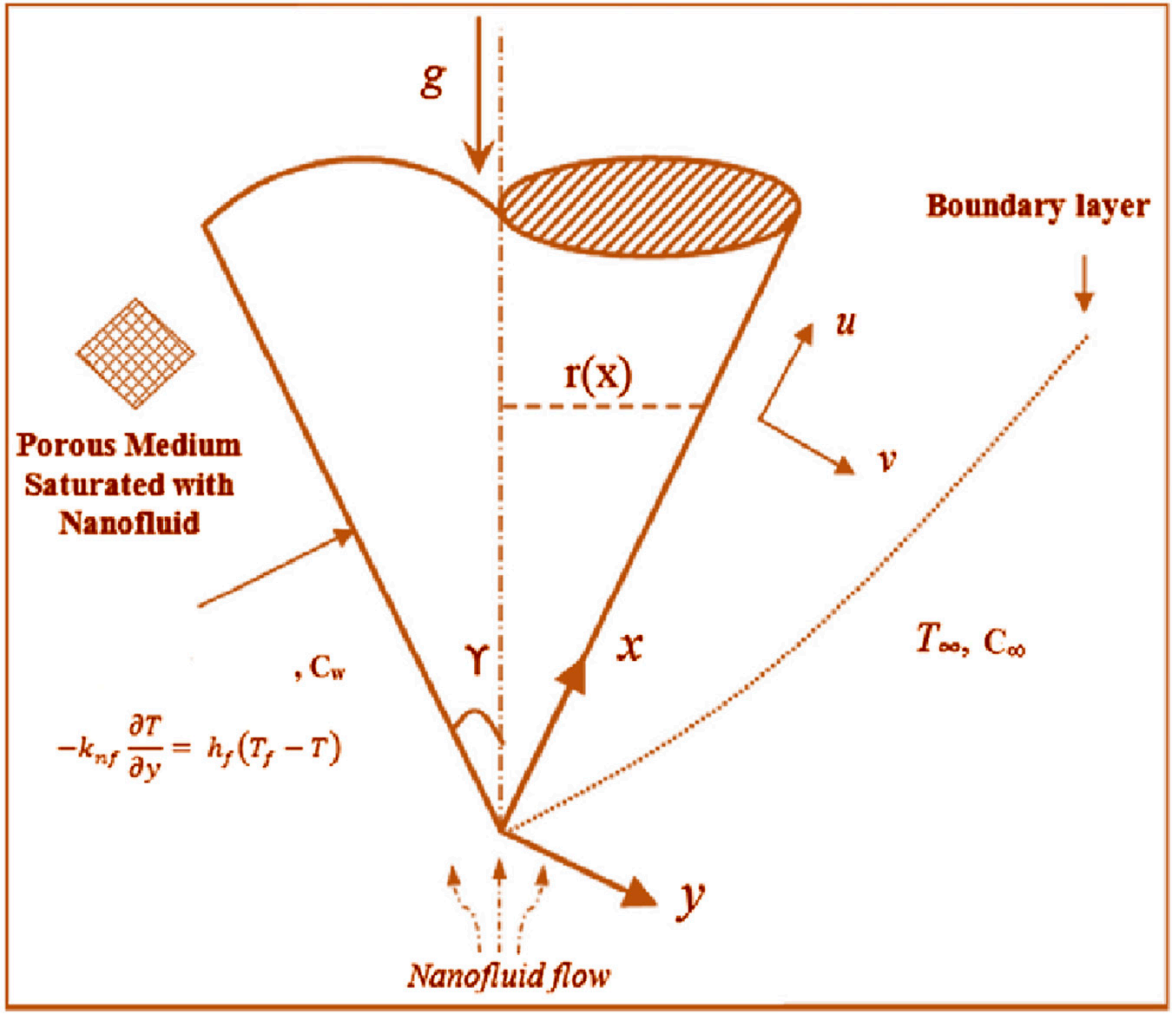
Flow geometry.

Laminar flow is studied in two dimensions across a porous vertical cone containing single and multiple CNTs based on an aqueous nanofluid movement with anomalous thermal performance (Figure 1). Table 1 demonstrates the thermophysical features of the base fluid (water) and CNTs. The x-axis of the selected geometry coincides with the path of flow through the cone’s surface. The unknown temperature $T_{w}$ is due to convective warming process with properties such as the initial temperature $T_{f}$ and the heat transfer coefficient $h_{f}$ , where $\phi_{w}$ is the NVF at the cone’s surface, $T_{\infty }$ is the heat of the ambient fluid, and $\phi_{\infty }$ is the NVF of the ambient fluid. The y-axis is subject to the magnetic field.

**TABLE 1:** Thermo-properties of nanofluid.

Fluid	$\rho \left(\frac{kg}{m^{3}}\right)$	$C_{p}\left(\frac{J}{kgK}\right)$	$k\left(\frac{W}{mk}\right)$
Base fluid	997.1	4,179	0.613
SWCNTs	2,600	425	6,600
MWCNTs	1,600	796	3,000

The leading flow equations in the occurrence of chemical, suction, injection, radiation, porous matrix, and heat mass transfer are described in Equations 1–6 by, Amrollahi et al. (2010), Rashmi et al. (2011), Pakdamana et al. (2012), Wang et al. (2013), and Akbar et al. (2023a):

$$\frac{\partial \left(ru\right)}{\partial x}+\frac{\partial \left(rv\right)}{\partial y}=0,$$ (1)

$$u\frac{\partial u}{\partial x}+v\frac{\partial u}{\partial y}=\frac{\mu_{nf}}{\rho_{nf}}\frac{\partial^{2}u}{\partial y^{2}}-\frac{\mu_{nf}}{\rho_{nf}}\frac{1}{K}u+g\left[\beta \left(T-T_{\infty }\right)-\beta^{*}\left(\phi -\phi_{\infty }\right)\right]\mathrm{Cos}\;\gamma -\frac{\sigma B_{0}^{2}}{\rho_{nf}}u,$$ (2)

$$u\frac{\partial T}{\partial x}+v\frac{\partial T}{\partial y}=\alpha_{nf}\frac{\partial^{2}T}{\partial y^{2}}-\frac{1}{{\left(\rho c_{p}\right)}_{nf}}\frac{\partial q_{r}}{\partial y},$$ (3)

$$u\frac{\partial \phi }{\partial x}+v\frac{\partial \phi }{\partial y}=D_{m}\frac{\partial^{2}\phi }{\partial y^{2}}-K_{r}\left(\phi -\phi_{\infty }\right),$$ (4)

with boundary conditions

$$u=0,v=V_{1}\left(x\right),-k_{nf}\frac{\partial T}{\partial y}=h_{f}\left(T_{f}-T\right),\phi =\phi_{w}\text{ at }y=0,$$ (5)

$$u\to 0,T\to T_{\infty },\phi \to \phi_{\infty }\text{ at }y\to \infty ,$$ (6)

qr=−16T∞σ3*3K*∂T∂y. (7)

Here, u and v signify the velocity components alongside the coordinate axes x and y, respectively. The expression $V0=\frac{3a}{4x}\;Ra_{x}^{1/4}v1$ denotes the mass transportation along the surface with suction $\left(v1<0\right)$ and injection $\left(v1>0\right)$ .

The thermal boundary conditions applied to a surface or contact in a system where convection is the primary mode of heat transmission are known as “convective boundary conditions”. The movement of a fluid and a solid surface to transfer heat is the main concern in setting a convective boundary condition. A common description of convective heat transport at a boundary is provided by Newton’s law of cooling.

Here, $T^{4}$ is expressed in terms of a Taylor sequence as follows:

$$T^{4}=T_{\infty }^{4}+4T_{\infty }^{3}\left(T-T_{\infty }\right)+6T_{\infty }^{2}{\left(T-T_{\infty }\right)}^{2}+\ldots$$

Neglecting higher-order terms, we derive

T4≅4T∞T3−3T∞4. (8)

By substituting Equation 8 in Equation 7, We get, Equation 9,

qr=−16T∞σ3*3K*∂T∂y. (9)

Thermal conductivity $k_{nf}$ is expressed as follows:

$$\mu_{nf}=\frac{\mu_{f}}{{\left(1-\varphi \right)}^{2.5}},\rho_{nf}=\left(1-\varphi \right)\rho_{f}+\rho_{CNT}\varphi ,\alpha_{nf}=\frac{k_{nf}}{{\left(\rho c_{p}\right)}_{nf}},{\left(\rho c_{p}\right)}_{nf}=\left(1-\varphi \right){\left(\rho c_{p}\right)}_{f}+\varphi {\left(\rho c_{p}\right)}_{CNT},k_{nf}=k_{f}\left(\frac{\left(1-\varphi \right)+2\varphi \left(\frac{k_{CNT}}{k_{CNT}-k_{f}}\right)\ln\;\left(\frac{k_{CNT}+k_{f}}{2k_{f}}\right)}{\left(1-\varphi \right)+2\varphi \left(\frac{k_{f}}{k_{CNT}-k_{f}}\right)\ln\;\left(\frac{k_{CNT+}+k_{f}}{2k_{f}}\right)}\right).$$

Introducing the similarity transformations:

$$\eta =\frac{y}{x}Ra_{x}^{1/4},f\left(\eta \right)=\frac{\psi }{\alpha Ra_{x}^{1/4}},\theta \left(\eta \right)=\frac{T-T_{\infty }}{T_{w}-T_{\infty }},S\left(\eta \right)=\frac{\phi -\phi_{\infty }}{\phi_{w}-\phi_{\infty }},$$ (10)

where $Ra_{x}$ is the local Rayleigh number and is defined as

$$Ra_{x}=\frac{g\beta_{bf}\rho_{bf}\left(T-T_{\infty }\right)x^{3}\mathrm{Cos}\;\gamma }{\mu_{bf}\alpha_{bf}}$$

$$f^{\prime \prime \prime }+\frac{A_{1}}{\mathrm{Pr}}\left[\frac{3}{4}ff^{\prime \prime }-\frac{1}{2}{\left(f^{\prime }\right)}^{2}\right]-k1f^{\prime }-\frac{A_{1}}{A_{2}}Mf^{\prime }+A_{1}\left[\theta -NrS\right]=0,$$ (11)

$$\left(1+R\right)\theta^{\prime \prime }+\frac{3}{4}A_{3}A_{4}f\theta^{\prime }=0,$$ (12)

$$S^{\prime \prime }+\frac{3}{4}ScfS^{\prime }-Cr.S=0.$$ (13)

The corresponding boundary constraints are

$$\begin{array}{c} \eta =0,f=V_{0},f^{\prime }=0,\theta^{\prime }\left(0\right)=-A_{4}B1\left(1-\theta \left(0\right)\right),S=1.\\ \eta \to \infty ,f^{\prime }=0,\theta =0,S=0. \end{array}$$ (14)

Prime signifies a derivative with respect to η , and the parameters defined in the above equations are

Nr=β*ϕw−ϕ∞βTw−T∞,k1=x2KRax1/2,Cr=Krx2DmRax1/2,Pr=μfαρf,R=16T∞σ3*3K*knf,M=σβo2x2μfRax1/2,Sc=αDm,A1=1−φ2.51−φ+φρCNTρf,B1=hfxkfRax14,A2=1−φ+φρCNTρf,A3=1−φ+φρCpCNTρCpf,A4=kfknf.

The physical quantities of interests are Equation 15,

$$C_{f}=\frac{\tau_{w}}{\rho U_{\infty }^{2}},Nu_{x}=\frac{xq_{w}}{k\left(T_{w}-T_{\infty }\right)},Sh_{x}=\frac{xJ_{w}}{D_{B}\left(\phi_{w}-\phi_{\infty }\right)}.$$ (15)

## 3 RK4 solution and validation

The numerical solution was obtained for the modeled mathematical problem (17)–(20) via fourth-order Runge–Kutta (RK4). The first order ordinary differential equations are obtained in dimensionless form by using the new variables. The boundary conditions are transformed as well. The Prandtl number is fixed as 6.2 for the water, as the model is water-based SWCNTs and MWCNTs. The value of NVF varies 0.1–0.3 ( 0<ϕ≤0.3 ). For conformation, the present study was related to previous analysis, and good correspondence was recognized by comparing the values of the skin friction (Table 2). This validation confirmed that that the numerical fallout of the existing research is definite.

**TABLE 2:** Confirmation of the present study by comparison of $f^{\prime \prime }\left(0\right)$ with Amrollahi et al. (2010) taking water as the base fluid when *Nr* = 0, *Pr* = 6.2, *V*0 = 0, *B*1 = 0, *γ* = 0, *R* = 0, *Cr* = 0, *M* = 0, *K*1 = 0.

φ	$f^{\prime \prime }\left(0\right)$			
	Khan et al. (Amrollahi et al., 2010)		Current work	
	SW-CNTs	MW-CNTs	SW-CNTs	MW-CNTs
0.1	0.33894	0.33727	0.33712	0.33736
0.2	0.40811	0.39008	0.40821	0.39001
0.3	0.50452	0.46466	0.50458	0.46344

## 4 Stability analysis

We are here interested in obtaining the second solution for the various values of λ . For this determination, it is important to determine the immovability examination of the problem which is reliable as well as actually realistic. The method approved is by Merkin (1986) as improved by Weidman et al. (2006). The unsteady flow equations are Equation 16,

$$\frac{\partial \left(ru\right)}{\partial x}+\frac{\partial \left(rv\right)}{\partial y}=0,$$ (16)

$$\frac{\partial u}{\partial t}+u\frac{\partial u}{\partial x}+v\frac{\partial u}{\partial y}=\frac{\mu_{nf}}{\rho_{nf}}\frac{\partial^{2}u}{\partial y^{2}}-\frac{\mu_{nf}}{\rho_{nf}}\frac{1}{K}u+g\left[\beta \left(T-T_{\infty }\right)-\beta^{*}\left(\phi -\phi_{\infty }\right)\right]\mathrm{Cos}\;\gamma -\frac{\sigma B_{0}^{2}}{\rho_{nf}}u,$$ (17)

$$\frac{\partial T}{\partial t}+u\frac{\partial T}{\partial x}+v\frac{\partial T}{\partial y}=\alpha_{nf}\frac{\partial^{2}T}{\partial y^{2}}-\frac{1}{{\left(\rho c_{p}\right)}_{nf}}\frac{\partial q_{r}}{\partial y},$$ (18)

$$u\frac{\partial \phi }{\partial t}+\frac{\partial \phi }{\partial x}+v\frac{\partial \phi }{\partial y}=D_{m}\frac{\partial^{2}\phi }{\partial y^{2}}-K_{r}\left(\phi -\phi_{\infty }\right),$$ (19)

with boundary conditions:

$$u=0,v=V_{1}\left(x\right),-k_{nf}\frac{\partial T}{\partial y}=h_{f}\left(T_{f}-T\right),\phi =\phi_{w}\text{ at }y=0,$$ (20)

$$u\to 0,T\to T_{\infty },\phi \to \phi_{\infty }\text{ at }y\to \infty .$$ (21)

The new similarity transformation is defined by introducing new parameter τ :

$$\eta =\frac{y}{x}Ra_{x}^{\frac{1}{4}},f\left(\eta ,\tau \right)=\frac{\psi }{\alpha Ra_{x}^{\frac{1}{4}}},\theta \left(\eta ,\tau \right)=\frac{T-T_{\infty }}{T_{w}-T_{\infty }},S\left(\eta ,\tau \right)=\frac{\phi -\phi_{\infty }}{\phi_{w}-\phi_{\infty }},\tau =ct.$$ (22)

Using Equation 22 in Equations 17–21, we obtain

$$\frac{\partial^{3}f}{\partial^{3}\eta }+\frac{A_{1}}{\mathrm{Pr}}\left[\frac{3}{4}f\frac{\partial^{2}f}{\partial^{2}\eta }-\frac{1}{2}{\left(\frac{\partial f}{\partial \eta }\right)}^{2}\right]-k1\frac{\partial f}{\partial \eta }-\frac{\partial^{2}f}{\partial \eta \partial \tau }-\frac{A_{1}}{A_{2}}M\frac{\partial f}{\partial \eta }+A_{1}\left[\theta -NrS\right]+\tau \left[\frac{\partial f}{\partial \tau }\frac{\partial^{2}f}{\partial^{2}\eta }-\frac{\partial f}{\partial \eta }\frac{\partial^{2}f}{\partial \eta \partial }\right]=0,$$ (23)

$$\left(1+R\right)\frac{\partial^{2}\theta }{\partial^{2}\eta }+\frac{3}{4}A_{3}A_{4}f\frac{\partial \theta }{\partial \eta }-\frac{3}{4}A_{3}A_{4}\left[\frac{\partial f}{\partial \tau }\frac{\partial \theta }{\partial \eta }-\frac{\partial f}{\partial \eta }\frac{\partial \theta }{\partial \tau }+\frac{\partial \theta }{\partial \tau }\right]=0,$$ (24)

$$\frac{\partial^{2}S}{\partial^{2}\eta }+\frac{3}{4}Scf\frac{\partial S}{\partial \eta }-CrS+\frac{\partial S}{\partial \tau }=0.$$ (25)

The corresponding boundary constraints are

$$f\left(0,\tau \right)=V_{0},\frac{\partial f}{\partial \eta }\left(0,\tau \right)=0,\frac{\partial \theta }{\partial \eta }\left(0,\tau \right)=-A_{4}B1\left(1-\theta \left(0,\tau \right)\right),S\left(0,\tau \right)=1,\text{as }\eta =0,\frac{\partial f}{\partial \eta }\left(\eta ,\tau \right)\to 0,\theta \left(\eta ,\tau \right)\to 0,S\left(\eta ,\tau \right)\to 0\text{ as }\eta \to \infty .$$ (26)

The stability explanation is attained by the steady movement $f\left(\eta \right)=f_{0}\left(\eta \right),\theta \left(\eta \right)=\theta_{0}\left(\eta \right),and\;S\left(\eta \right)=S_{0}\left(\eta \right)$ by expressing the following relations (Merkin, 1986; Weidman et al., 2006):

$$f\left(\eta ,\tau \right)=f_{0}\left(\eta \right)+e^{-\gamma \tau }J\left(\eta ,\tau \right),\theta \left(\eta ,\tau \right)=\theta_{0}\left(\eta \right)+e^{-\gamma \tau }F\left(\eta ,\tau \right),S\left(\eta \right)=S_{0}\left(\eta \right)+e^{-\gamma \tau }G\left(\eta ,\tau \right).$$ (27)

Here, γ is an undetermined eigenvalue and $J\left(\eta ,\tau \right)$ , $\theta \left(\eta ,\tau \right)$ , and $S\left(\eta ,\tau \right)$ are relatively smaller than $f_{0}\left(\eta \right),$ $\theta_{0}\left(\eta \right),$ and $S_{0}\left(\eta \right)$ .

Using Equation 27 and Equations 23–26, we obtain

$$\frac{\partial^{3}J}{\partial^{3}\eta }+\frac{A_{1}}{\mathrm{Pr}}\left[\frac{3}{4}f_{0}\frac{\partial^{2}J}{\partial^{2}\eta }+f_{0}^{\prime \prime }J-f_{0}^{\prime }f_{0}^{\prime \prime }\right]-k1\frac{\partial J}{\partial \eta }+\frac{\partial^{2}J}{\partial \eta \partial \tau }-\frac{A_{1}}{A_{2}}M\frac{\partial J}{\partial \eta }+A_{1}\left[F-NrG\right]+\gamma \left[\gamma f_{0}^{\prime \prime }\frac{\partial J}{\partial \tau }+\frac{\partial J}{\partial \tau }\frac{\partial^{2}J}{\partial^{2}\eta }+f_{0}^{\prime }\frac{\partial^{2}J}{\partial^{2}\eta }-f_{0}^{\prime }\frac{\partial^{2}J}{\partial \eta \partial \tau }\right]=0,$$ (28)

$$\left(1+R\right)\frac{\partial^{2}F}{\partial^{2}\eta }+\frac{3}{4}A_{3}A_{4}\left(f_{0}\frac{\partial F}{\partial \eta }-\theta_{0}^{\prime }J\right)-\frac{3}{4}A_{3}A_{4}\left[\gamma \theta_{0}^{\prime }J+\theta_{0}\frac{\partial J}{\partial \tau }-\gamma Ff_{0}-f_{0}\frac{\partial F}{\partial \tau }-\gamma F+\frac{\partial F}{\partial \tau }\right]=0,$$ (29)

$$\frac{\partial^{2}G}{\partial^{2}\eta }+\frac{3}{4}Sc\left(f_{0}\frac{\partial G}{\partial \eta }+S_{0}J\right)-CrG-\gamma G+\frac{\partial G}{\partial \tau }=0.$$ (30)

The corresponding boundary constraints are

$$J\left(0,\tau \right)=V_{0},\frac{\partial J}{\partial \eta }\left(0,\tau \right)=0,\frac{\partial F}{\partial \eta }\left(0,\tau \right)=-A_{4}B1\left(1-F\left(0,\tau \right)\right),G\left(0,\tau \right)=1,\frac{\partial J}{\partial \eta }\left(\eta ,\tau \right)\to 0,F\left(\eta ,\tau \right)\to 0,G\left(\eta ,\tau \right)\to 0.\text{ as }\eta \to \infty .$$ (31)

The steady state solution for $J\left(\eta \right)=J_{0}\left(\eta \right),F\left(\eta \right)=F_{0}\left(\eta \right),\text{and }G\left(\eta \right)=G_{0}\left(\eta \right)$ for stability analysis is obtained by setting τ=0 for the system Equations 11–14. Therefore, $f\left(\eta \right)=f_{0}\left(\eta \right),\theta \left(\eta \right)=\theta_{0}\left(\eta \right),\text{and }S\left(\eta \right)=S_{0}\left(\eta \right)$ in system Equations 28–31 signify the initial growth or decay of the solution given by Equation 27. Consequently, the linear eigenvalue problem is obtained thus:

$$J_{0}^{\prime \prime \prime }+\frac{A_{1}}{\mathrm{Pr}}\left[\frac{3}{4}f_{0}J_{0}^{\prime \prime }+f_{0}^{\prime \prime }J-f_{0}^{\prime }f_{0}^{\prime \prime }\right]-k1J_{0}^{\prime }-\frac{A_{1}}{A_{2}}MJ_{0}^{\prime }+A_{1}\left[F_{0}-NrG_{0}\right]+\gamma f_{0}^{\prime }J_{0}^{\prime \prime }=0,$$ (32)

$$\left(1+R\right)F_{0}^{\prime \prime }+\frac{3}{4}A_{3}A_{4}\left(f_{0}F_{0}^{\prime }-\theta_{0}^{\prime }J_{0}\right)-\frac{3}{4}A_{3}A_{4}\left[\gamma \theta_{0}^{\prime }J_{0}-\gamma F_{0}f_{0}-\gamma F_{0}\right]=0,$$ (33)

$$G_{0}^{\prime \prime }+\frac{3}{4}Sc\left(f_{0}G_{0}^{\prime }+S_{0}J_{0}\right)-CrG_{0}-\gamma G_{0}=0.$$ (34)

The corresponding boundary constraints are

$$J_{0}\left(0,\tau \right)=V1,J_{0}^{\prime }\left(0,\tau \right)=0,F_{0}^{\prime }\left(0,\tau \right)=-A_{4}B1\left(1-F_{0}\left(0,\tau \right)\right),G_{0}\left(0,\tau \right)=1,J_{0}^{\prime }\left(\eta ,\tau \right)\to 0,F_{0}\left(\eta ,\tau \right)\to 0,G_{0}\left(\eta ,\tau \right)\to 0,\text{as }\eta \to \infty .$$ (35)

## 5 Results and discussion

This study aims to investigate the impact of single-walled (SWCNTs) and multi-walled (MWCNTs) carbon nanotubes with convectional fluid (water) over a perpendicular cone beneath the influences of chemical species, magnetism, thermal radiation, and drenched permeable media. The impact of heat is also examined. Constructed on the flow norms, the fundamental flow characteristics are modeled in terms of partial differential equations (PDEs). Using the appropriate transformation, the PDEs are denationalized in terms of conventional derivatives (ordinary differential equations) and then solved via RK4 in MATLAB.

**FIGURE 2:**
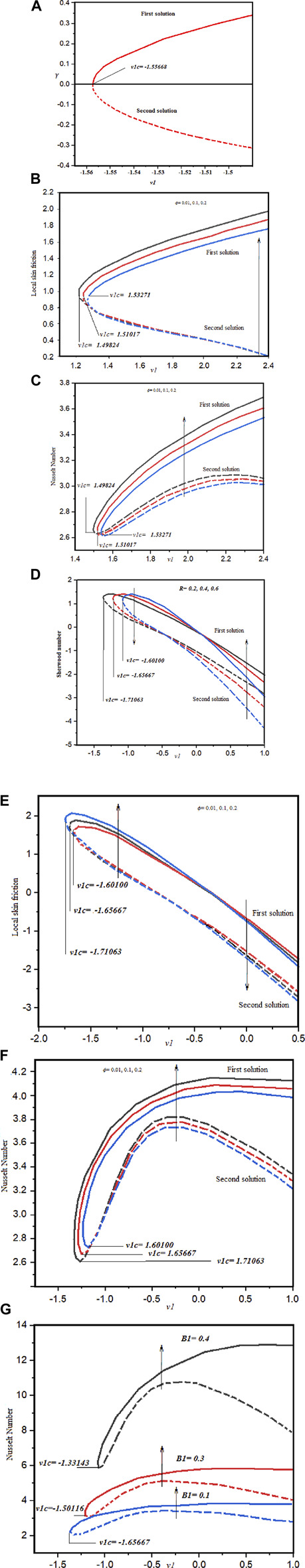
**(A)** Smallest eigenvalues γ with suction parameter v1<0 . **(B)** Local skin friction with injection $\left(v1>0\right)$ for ϕ . **(C)** Nusselt number with injection parameter $\left(v1>0\right)$ for ϕ . **(D)** Sherwood number for v1<0 and v1>0 using R . **(E)** Skin friction for v1<0 and v1>0 using ϕ . **(F)** Nusselt number for v1<0 and v1>0 using ϕ . **(G)** Nusselt number for *B1* v1<0 and v1>0 .

The mathematical framework given in Equations 10–13 with Equation 14 presented multiple solutions on behalf of some evolving constraints. Figure 2A demonstrates the sensations of double solutions—reliable (first solution) and unreliable (second solution). Consequently, an immovability examination is executed to determine a reliable solution. Equation 35, as well as linearized Equations 32–34, were numerically solved using RK4 MATLAB software. Figure 2A shows the lowest eigenvalues γ versus V1 when ϕ=0.01 . Subsequently, the suction parameter neared its critical value, and the values of γ propagated nearer to zero in both (first and second) solutions. We suggest that the first solution is stable whereas the second is unreliably constructed on the earlier exploration. This methodology is vital for finding a reliable solution whenever multiple solutions occur, permitting reliable movement-behavior extrapolation.

Figures 2B and C confirm the effects of the volume fraction parameter (VFP) verses suction factor v1 on local skin friction (LSF) and the Nusselt number (NN). In this analysis, duality is observed for the given values v1 . In the case of ϕ=0.01 , solutions are guaranteed to occur for v1c > 1.53271, but when ϕ= grows to 0.1 and 0.2 and v1c > 1.51017 and 1.49824, respectively, the range of suction expands. Additionally, it should be noted that increasing VFP enhances from 0.01 to 0.03, improving both LSF and the NN. Additionally, the values of LSF and the NN for the first solution rise when the suction parameter v1 rises, but the reverse occurs for the second solution.

The impact of radiation and volume fraction parameters on the Sherwood number and LSF is depicted in Figures 2D and E, respectively, along both suction and injection parameters. It is apparent that dual solutions occurred for both the Sherwood number and LSF. However, no solution exists for v1<v1c (critical point), demonstrating that the boundary layer splits from the cone surface and violates the rules of the boundary layer model. Additionally, v1c is the point of contact that combines the reliable and unreliable solutions. In Figure 2D, the Sherwood number is enhanced in the first and second solutions with increasing radiation parameter when v1>0 and declines when v1<0 . Figure 2E shows that LSF increases in both the first and second solutions for v1>0 and declines for v1<0 with growing values of VFP.

We found that the VFP increased the NN in both the first and second solutions (Figure 2F). It is significant that increasing the nanoparticles’ VFP enhances heat transportation efficiency. The influence of the Biot number on the NN is demonstrated in Figure 2G. Here, in both first and second solution, the NN increases with a higher Biot number.

**FIGURE 3:**
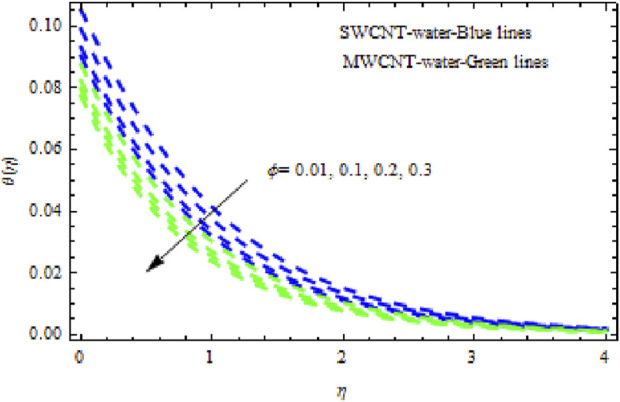
Impact of ϕ on $f^{\prime }\left(\eta \right).$

**FIGURE 4:**
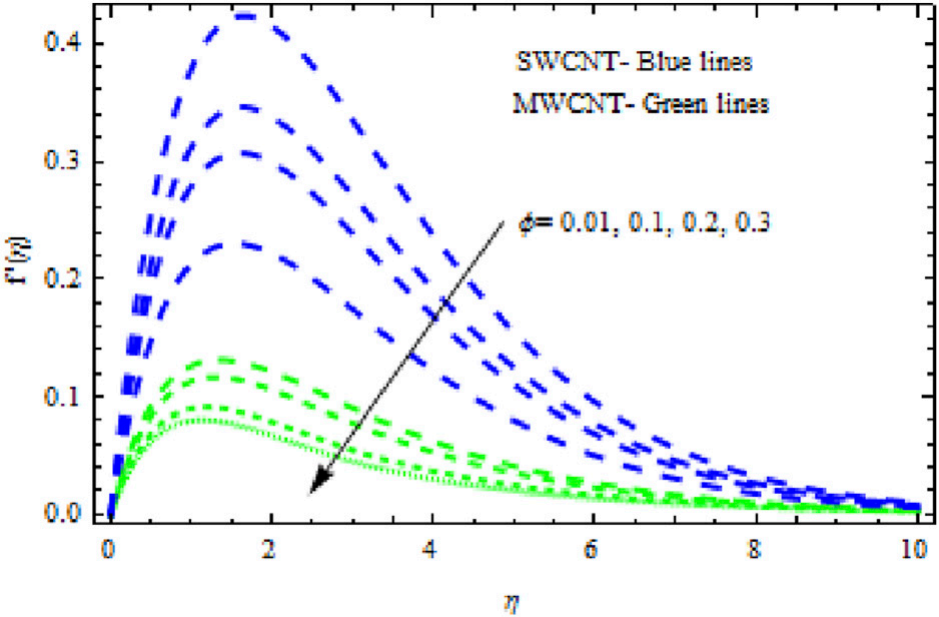
Impact of ϕ on $\theta \left(\eta \right).$

**FIGURE 5:**
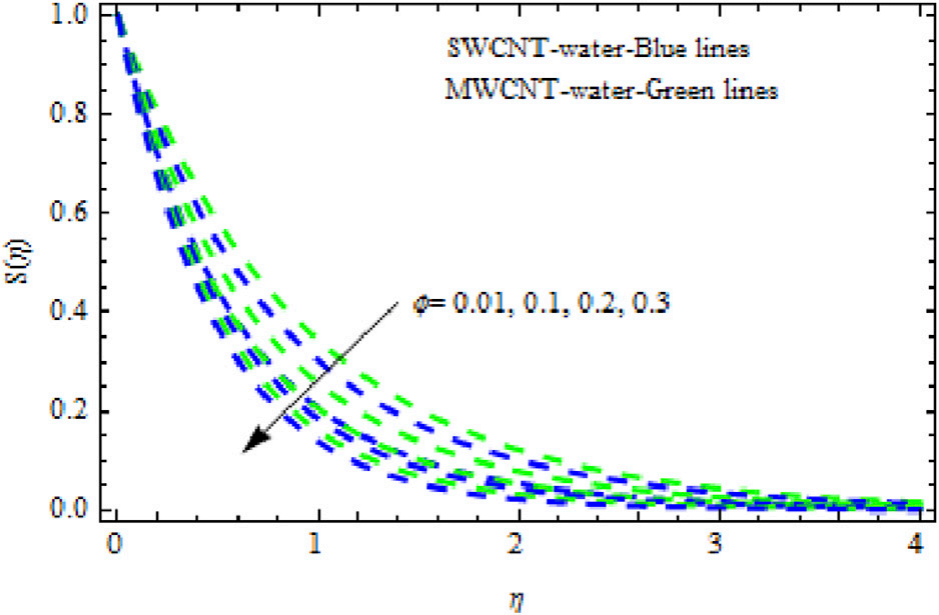
Impact of ϕ on $S\left(\eta \right).$

**FIGURE 6:**
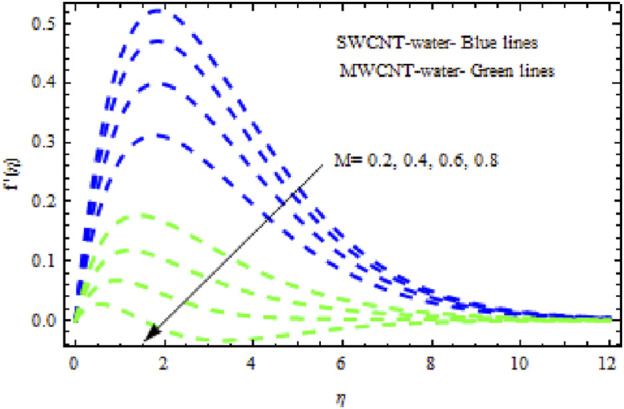
Impact of M on $f^{\prime }\left(\eta \right).$

**FIGURE 7:**
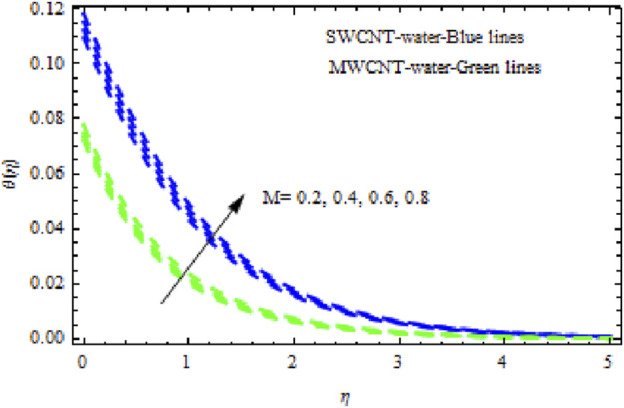
Impact of M on $\theta \left(\eta \right).$

**FIGURE 8:**
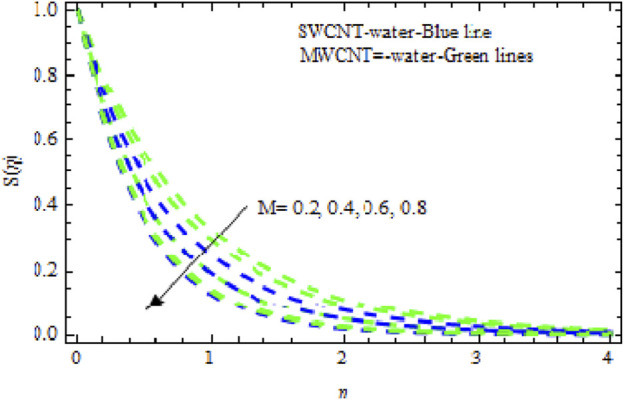
Impact of M on $S\left(\eta \right).$

**FIGURE 9:**
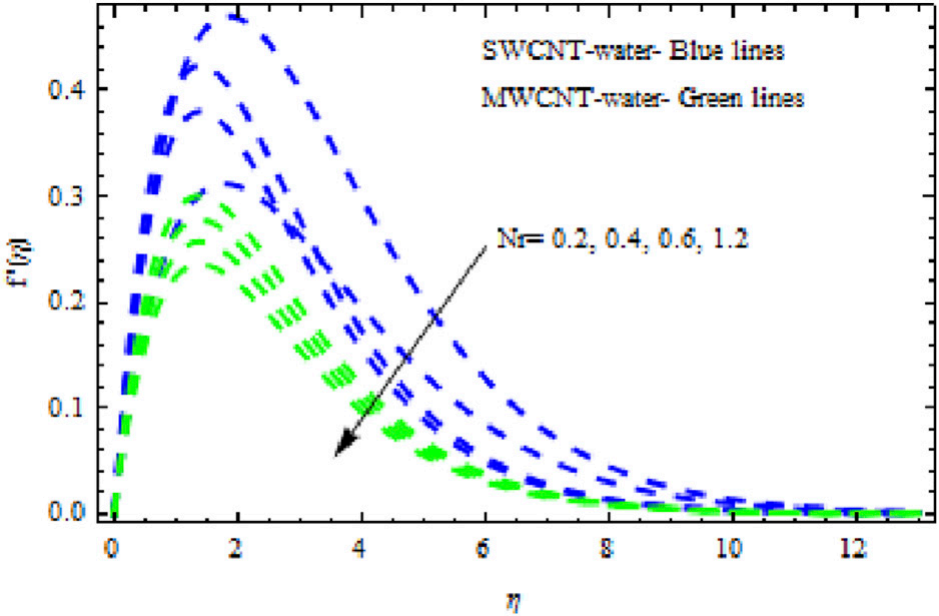
Impact of Nr on $f^{\prime }\left(\eta \right).$

**FIGURE 10:**
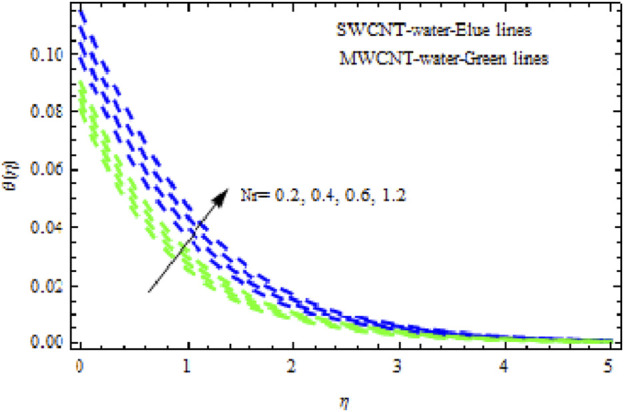
Impact of Nr on $\theta \left(\eta \right).$

**FIGURE 11:**
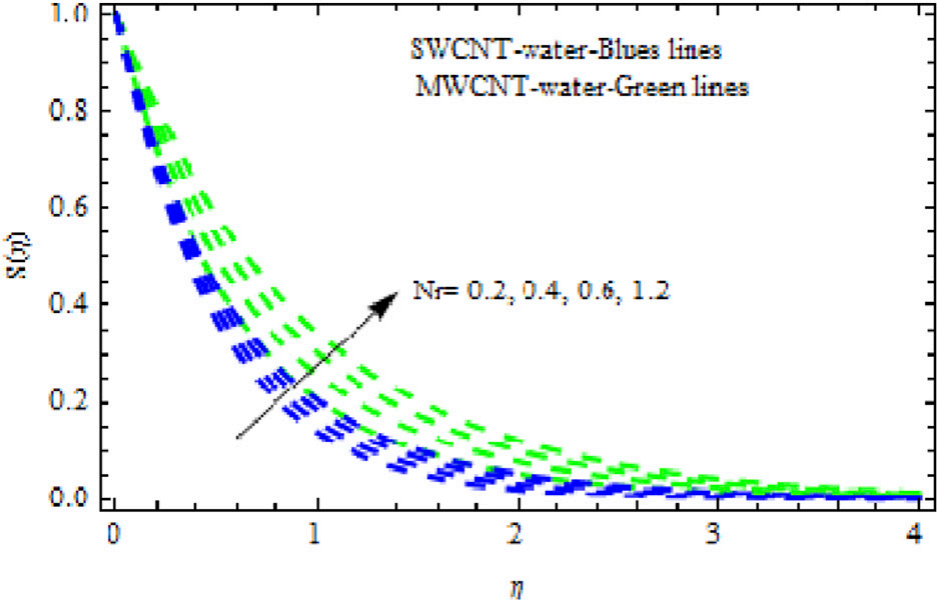
Impact of Nr on $S\left(\eta \right)$ .

**FIGURE 12:**
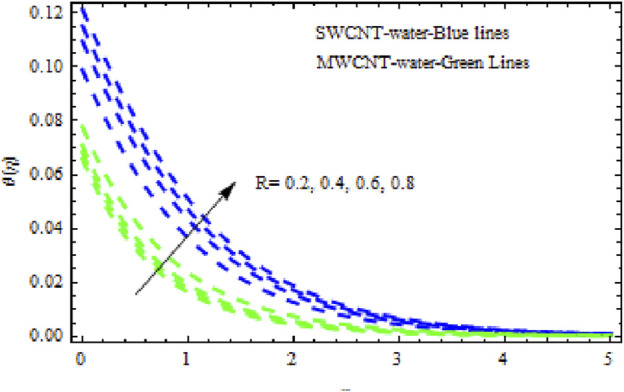
Impact of R on $\theta \left(\eta \right).$

**FIGURE 13:**
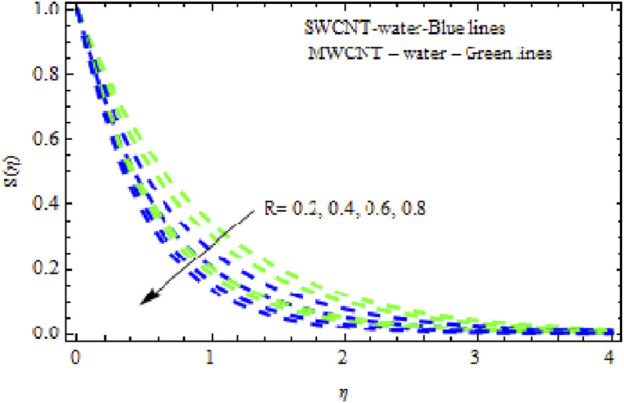
Impact of R on $S\left(\eta \right).$

**FIGURE 14:**
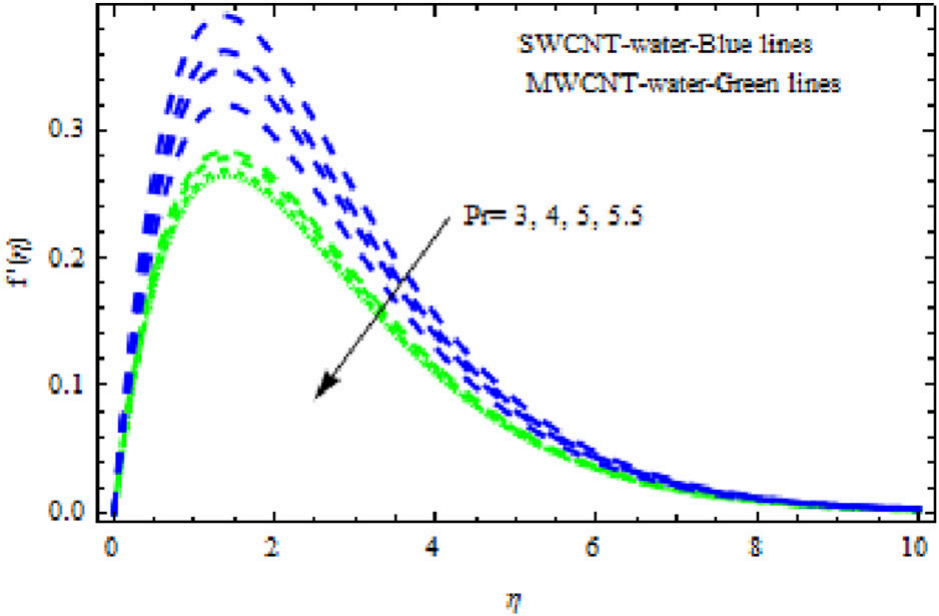
Impact of Pr on $f^{\prime }\left(\eta \right).$

**FIGURE 15:**
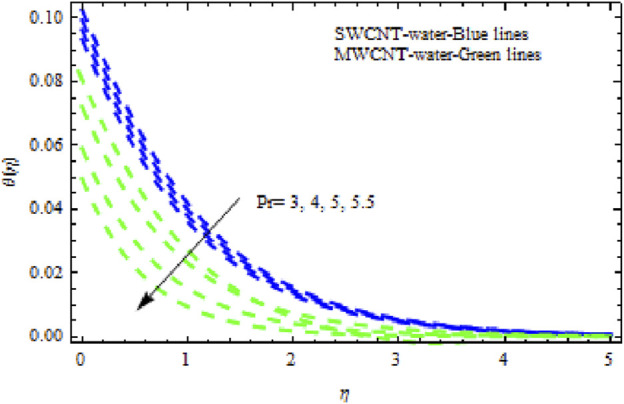
Impact of Pr on $\theta \left(\eta \right).$

**FIGURE 16:**
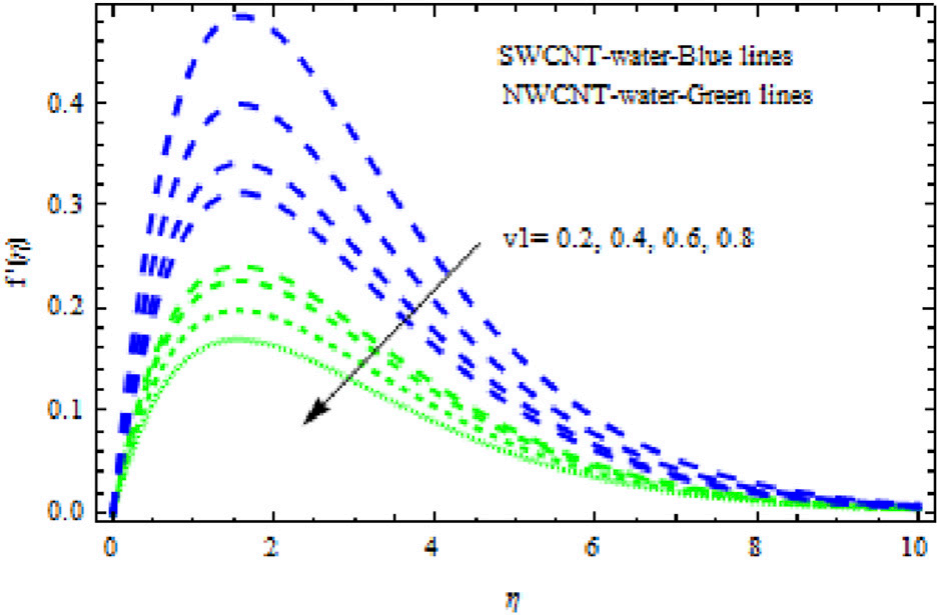
Impact of v1 on $f^{\prime }\left(\eta \right).$

**FIGURE 17:**
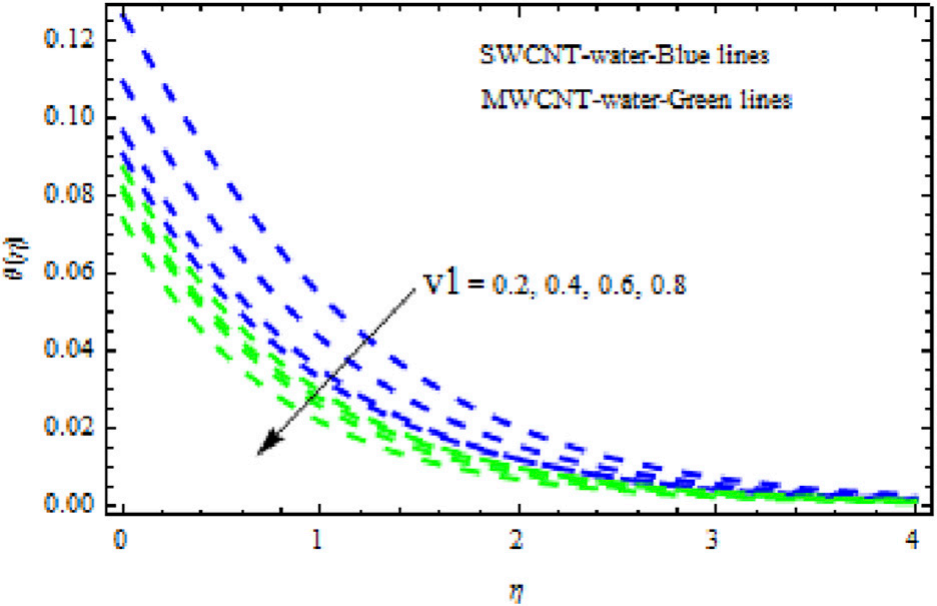
Influence of v1 on $\theta \left(\eta \right).$

**FIGURE 18:**
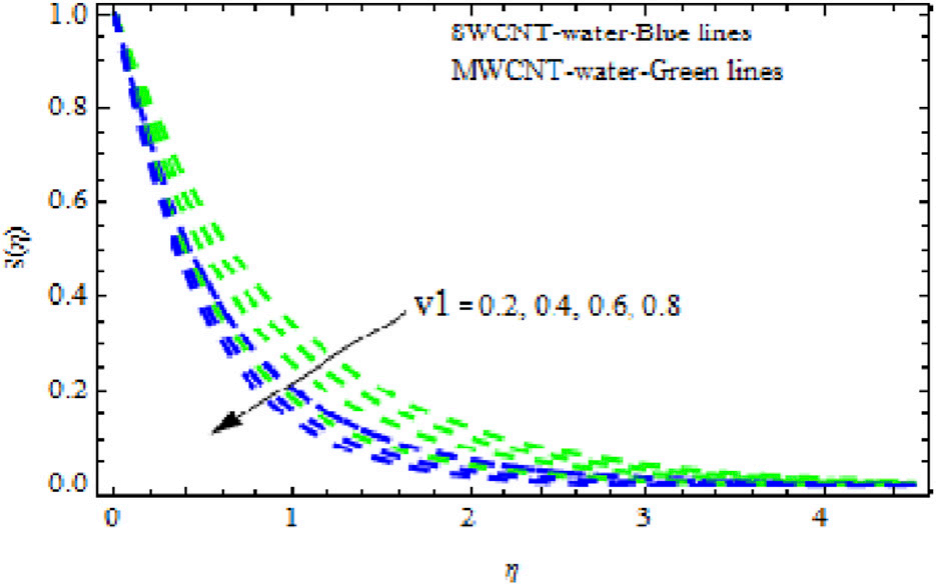
Impact of v1 on $S\left(\eta \right).$

**FIGURE 19:**
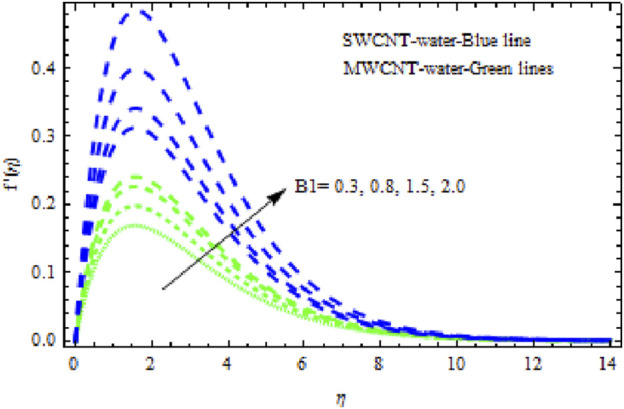
Impact of B1 on $f^{\prime }\left(\eta \right).$

**FIGURE 20:**
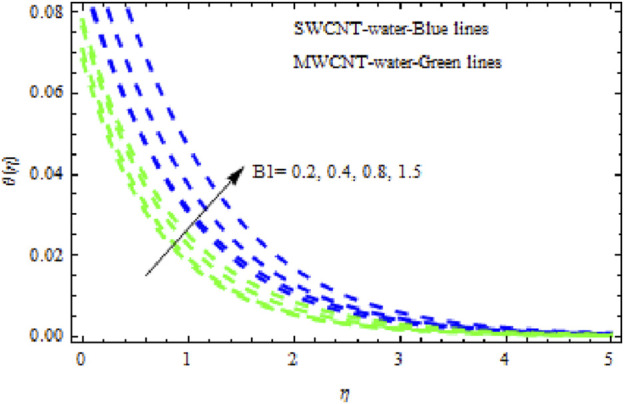
Impact of B1 on $\theta \left(\eta \right).$

**FIGURE 21:**
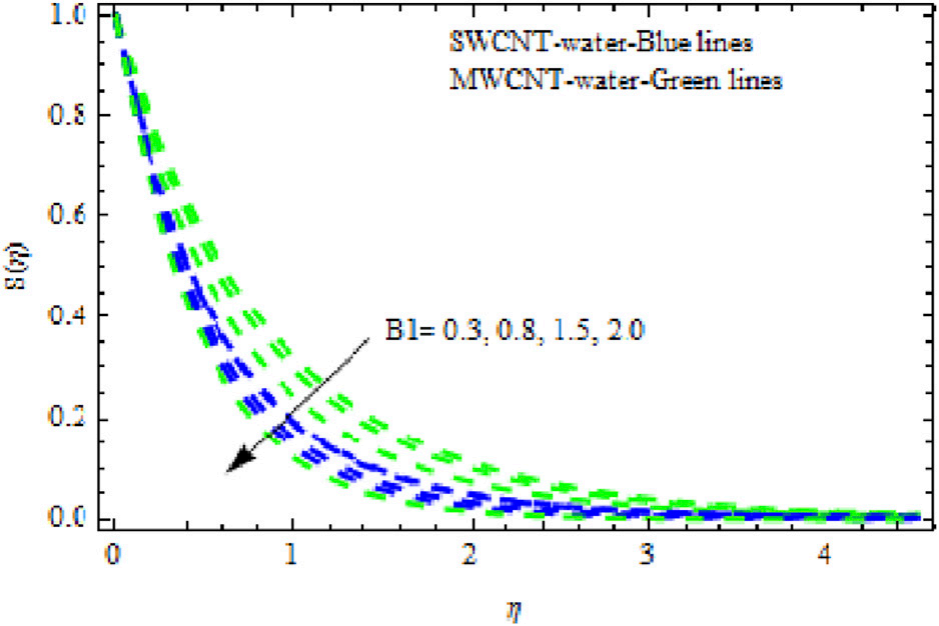
Influence of B1 on $S\left(\eta \right).$

**FIGURE 22:**
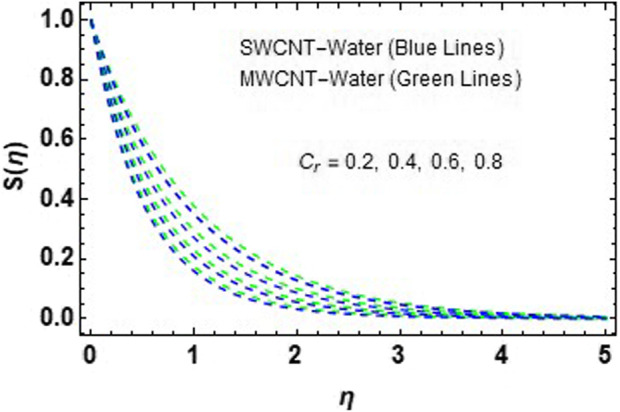
Impact of Cr on $S\left(\eta \right).$

**FIGURE 23:**
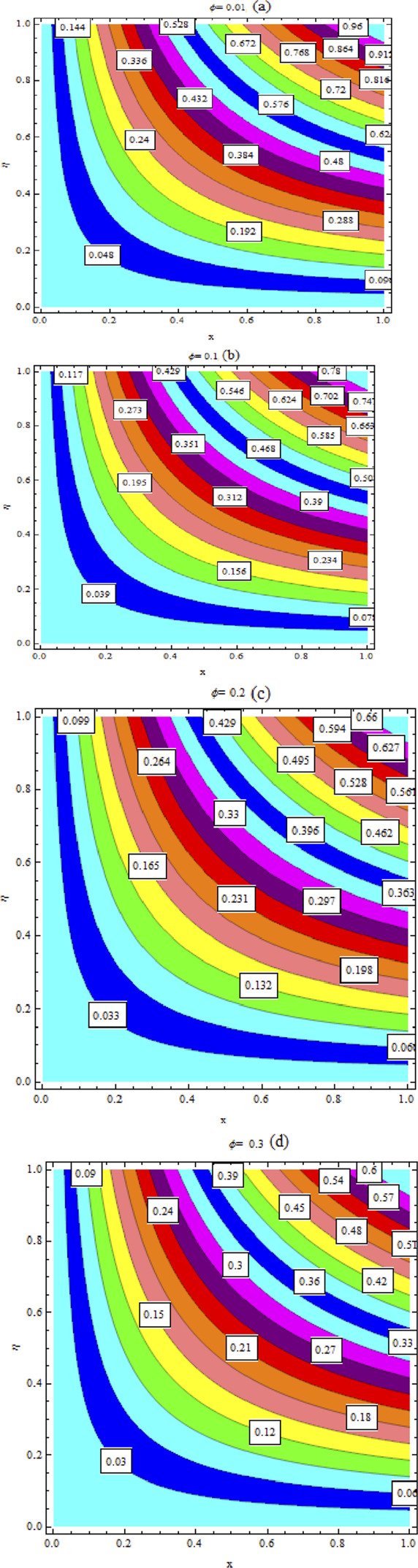
Streamlines for: **(A)** ϕ=0.01 ; **(B)** ϕ=0.1 ; **(C)** ϕ=0.2 ; **(D)** ϕ=0.3 .

The distributions of velocity, temperature, and concentration of nanofluid (NF) (water base), including SWCNTs and MWCNTs, for different values of VFP are shown in Figures 3–5. In both cases, the velocity outlines decreased as VFP increased. It is important to note that for MWCNTs, the velocity of the fluid is significantly degraded in comparison to the SWCNT-water-based NFs. As VFP increases, the humidity and concentration profiles of both NFs slow. We also found that MWCNTs demonstrate greater thermal boundary layer thickness (TBLT) retardation than SWCNTs. Nevertheless, SWCNT NF experiences a significantly greater decrease in solutal boundary layer width than MWCNT NF. The impact of magnetic factor $\left(M\right)$ is portrayed in Figures 6–8. Figure 6 shows that for both SWCNT and MWCNT NF, the velocity patterns are impeded across the boundary layer with increasing values of magnetic factor $\left(M\right).$ Because the Lorentz effect, due to applied magnetic field, serves as a resistive force, boundary layer movement decreases everywhere in the flow. With greater values of $\left(M\right)$ , the depth of the hydrodynamic boundary layer in MWCNT NF (water as base fluid) declines more quickly than SWCNT-NF (water as base fluid). Figure 7 displays that as the magnitude of $\left(M\right)$ grows, the temperature curves of both liquids rise but rises significantly in SWCNT NF compared to MWCNT NF. Figure 8 shows that when $\left(M\right)$ increases, the concentration curves move in the opposite direction of the temperature curves. In Figures 9–11, we see how varying the Buoyancy ratio parameter $\left(Nr\right)$ affects the flow characteristics. As illustrated in Figure 9, both NFs experience a significant slowing in mobility with increasing levels of $\left(Nr\right).$ It was detected that with larger values of $\left(Nr\right),$ the temperature curves for the two NFs become more complex. This is because the thermal boundary layer thickness rises with growing $\left(Nr\right)$ (Figure 10). Likewise, the concentration characteristics rise with increasing (*Nr*). Figure 11 shows that the concentration patterns of MWCNT NFs rise more highly than those of SWCNT NFs. Figures 12, 13 show the temperature and concentration variations in the SWCNT and MWCNT NFs for different variations of radiation parameter (R). It is observed for both NFs that the thermal-boundary depth is enriched when R increases. This is because when R is enhanced, the Rosseland radiated absorptive k is too. This occurs more highly due to the rate of heating being submerged, denoted by ${\partial q}_{r}/\partial y$ . Enhanced radiative heat transmission promotes the expansion of the thermal boundary layer. However, when R grows, the concentration profiles become less robust in the fluid area. This slowdown in the thickness of the solutal boundary layer is thought to be more pronounced in MWCNT NF than in SWCNT NF. The consequence of the Prandtl number $\left(\mathrm{Pr}\right)$ on fluid flow and heat curves is seen in Figures 14, 15. As shown in Figure 14, MWCNT NF experiences a greater decrease in velocity distributions over the entire boundary layer regime than SWCNT NF when the value of Pr increases. In addition, higher concentrations of Pr in both NFs lead to lower temperature distributions. Physically, a higher Prandtl number indicating lower thermal diffusivity signifies the smallest heat diffusion rate in the fluid. Consequently, as shown in Figure 15, heat and the thermal boundary layer decline. Furthermore, deceleration is greater in MWCNT NF than in SWCNT NF. In Figures 16–18, we see how dimensionless velocity, temperature, and concentration vary with differing suction/injection factors ( v1 ). With increasing values of $v1\left(>0\right)$ , it appears that all of the curves degrade across the boundary layer zone for both NFs. This is because suction removes heated fluid through the boundary layer regime, causing a decrease in the thickness of all boundary layers. Additionally, MWCNT NF exhibits greater degradation in temperature and flow rate than SWCNT NF. The latter, however, exhibits decreasing concentration distributions at a greater rate than MWCNT NF (Figure 18). Figures 19–21 show how the Biot number $\left(B1\right)$ affects the momentum, temperature, and solute boundary layers for the two NFs. Figures 19 and 20 show that the flow rate and temperature of the liquid noticeably increase, and this rise is higher in SWCNT NFs than in MWCNT NFs. Physically, when B1 is augmented, the inner thermal force increases relative to the thermal boundary layer, raising the fluid’s heat in the boundary layer regime. However, when B1 values increase in intensity, the concentration profiles of the two NFs decrease (Figure 21). For SWCNT NF and MWCNT NFs, the solutal boundary layer is plotted in Figure 22 with varied values of the chemical reaction factor $\left(Cr\right)$ . It has been noted that MWCNT NFs exhibit significantly more degradation in concentration curves than SWCNT NFs. Streamlines are plotted in Figures 23A–D.

## 6 Conclusion

We here examine the heat transfer behavior and chemical interactions on the movement of water-based nanofluids (NFs) including single- (SWCNT) and multiple- walled (MWCNT) carbon nanotubes (CNTs) across an upward cone inserted into a porous material under a convection boundary constraints. The system of ordinary differential equations is numerically solved via RK4. Hydrodynamic, thermal, and solutal boundary layers are studied, and the effects of varying relevant parameters are shown graphically. The present analysis reveals the dual solution for the suction and injection parameter, and stability analysis is performed to confirm which solution is stable. The aim of this study is to analyze SWCNTs and MWCNTs in a vertical cone with stability to established that only the first solution is reliable. A table presentation displays the outcomes of LSF, heat transfer rate, NN, SN, and concentration. It is found that in both nanofluids, the heat transmission rate is a growing function of ϕ . Interestingly, the heat transmission rate is sophisticated in MWCNT water-based NF. The fluid rate diminishes with the growing magnitude of the magnetic factor $\left(M\right)$ while heat is enhanced with M. Heat mass transportation increases in both NFs with the augmented values of v1>0. This increase is higher in MWCNT NF. The temperature curves are augmented with the growing values of B1 .

### 6.1 Future suggestions

This model’s usefulness is rather limited because it depends solely on a two-dimensional flow arrangement that includes conventional thermal and mass boundary circumstances. Still, the inquiry would be useful for engineering purposes if conducted in three dimensions via the passive control technique and includes a convection heating/melting heat exchange mechanism along with stability assentation.

## Data Availability

The original contributions presented in the study are included in the article/supplementary material; further inquiries can be directed to the corresponding authors.
